# Impact of Genotype Imputation on the Performance of GBLUP and Bayesian Methods for Genomic Prediction

**DOI:** 10.1371/journal.pone.0101544

**Published:** 2014-07-15

**Authors:** Liuhong Chen, Changxi Li, Mehdi Sargolzaei, Flavio Schenkel

**Affiliations:** 1 Department of Agricultural, Food and Nutritional Science, University of Alberta, Edmonton, Alberta, Canada; 2 Agriculture and Agri-Food Canada, Lacombe Research Centre, Lacombe, Alberta, Canada; 3 Department of Animal and Poultry Science, University of Guelph, Guelph, Ontario, Canada; 4 L'Alliance Boviteq Inc., Quebec, Quebec, Canada; China Agricultrual University, China

## Abstract

The aim of this study was to evaluate the impact of genotype imputation on the performance of the GBLUP and Bayesian methods for genomic prediction. A total of 10,309 Holstein bulls were genotyped on the BovineSNP50 BeadChip (50 k). Five low density single nucleotide polymorphism (SNP) panels, containing 6,177, 2,480, 1,536, 768 and 384 SNPs, were simulated from the 50 k panel. A fraction of 0%, 33% and 66% of the animals were randomly selected from the training sets to have low density genotypes which were then imputed into 50 k genotypes. A GBLUP and a Bayesian method were used to predict direct genomic values (DGV) for validation animals using imputed or their actual 50 k genotypes. Traits studied included milk yield, fat percentage, protein percentage and somatic cell score (SCS). Results showed that performance of both GBLUP and Bayesian methods was influenced by imputation errors. For traits affected by a few large QTL, the Bayesian method resulted in greater reductions of accuracy due to imputation errors than GBLUP. Including SNPs with largest effects in the low density panel substantially improved the accuracy of genomic prediction for the Bayesian method. Including genotypes imputed from the 6 k panel achieved almost the same accuracy of genomic prediction as that of using the 50 k panel even when 66% of the training population was genotyped on the 6 k panel. These results justified the application of the 6 k panel for genomic prediction. Imputations from lower density panels were more prone to errors and resulted in lower accuracy of genomic prediction. But for animals that have close relationship to the reference set, genotype imputation may still achieve a relatively high accuracy.

## Introduction

Genomic selection has become a new tool for genetic improvement in livestock species and plants thanks to the discovery of many thousands of single nucleotide polymorphisms (SNP) and cost-effective high-throughput genotyping technology. Since the first publication of Meuwissen et al. [1], numerous statistical methods have been proposed for genomic prediction. Two main categories are genomic best linear unbiased prediction (GBLUP) methods [2]–[7], and Bayesian methods [1], [8]–[10].

Assumptions of SNP marker effects on the trait vary across different statistical methods. In general, GBLUP methods assume that all markers effects are from a normal distribution, while Bayesian methods assume non-normally distributed marker effects with distributions vary in different methods. Performance of different methods depends on the genetic architecture underlying the studied trait [11]. For traits that are affected by a few large quantitative trait loci (QTL), Bayesian methods usually outperform the GBLUP method, while for traits that are affected by many QTL with small effects, GBLUP would likely perform better than or similar as the Bayesian methods [11]–[13].

Accuracy in estimated breeding values has been increased significantly for young candidates in dairy cattle from application of genomic prediction using the BovineSNP50 BeadChip (50 k; Illumina Inc., San Diego, USA) [12], [13]. While the current price of genotyping on the 50 k panel still hurdles a large number of male and female candidates to be screened, a cost-effective strategy has been proposed that uses cheap low density panels for genotyping followed by imputing to the 50 k panel [14]–[17]. In October 2011, Illumina, Inc. has released the BovineLD Genotyping BeadChip (6 k) to replace its predecessor, the Illumina Golden Gate Bovine3K chip (3 k). Compared to the 3 k panel, the 6 k panel has lower genotyping errors, denser genome coverage, and more accuracy in imputing to 50 k genotypes [14], [18]. The GeneSeek Genomic Profiler (GGP) BeadChip has also become available since February 2012. The GGP chip has slightly higher imputation accuracy than the 6 k panel but performs similar as the 6 k panel in genomic evaluations [19]. The Illumina Golden Gate technology also allows an array to be customized containing 96, or from 384 to 3072 SNP loci. Genotype imputation from these lower density panels to 50 k genotypes was poorer than those from the 3 k, 6 k or the GGP chip. But these lower density panels may still have their applicability for genotyping selection candidates whose both parents have genotypes with at least one genotyped on the 50 k panel [16], [20]. The cheap, cost-effective low density panels and high accuracy of genotyping imputation would facilitate genomic selection by allowing screening on a larger number of young bulls and allowing for the selection to be conducted on cows.

Studies have shown that imputation errors affect the accuracy of genomic prediction [21], [22]. However, no comparisons of the impact of imputation errors on different genomic prediction methods have been reported. The aim of this study was to evaluate the performance of the GBLUP and Bayesian methods when imputed genotypes were used for genomic prediction.

## Materials and Methods

### Genotypes

A total of 10,309 Holstein dairy bulls born between 1950 and 2007 were genotyped on the Illumina BovineSNP50 BeadChip (50 k; Illumina Inc., San Diego, USA). SNPs with minor allele frequency (MAF) less than 0.05, missing rate more than 15% or P-value from Hardy-Weinberg disequilibrium test smaller than 0.0001 were filtered. After filtering, 35,790 SNPs with known locations on autosomal chromosomes were kept for analyses.

Five low density SNP panels including the Illumina Golden Gate Bovine3K BeadChip (3 k), the BovineLD BeadChip (6 k), and three simulated panels containing 384 (L384), 768 (L768), and 1536 (L1536) SNPs were considered in this study. In fact, 2,480 and 6,177 SNPs, from the 3 k and 6 k panel, respectively, coincided with the 50 k panel. Therefore, genotypes of the 2,480 and 6,177 SNPs extracted from the 50 k panel were used instead of re-genotyping animals on the 3 k and 6 k panels. The size of the simulated panels was chosen according to Illumina Golden Gate technology, which allows customized genotyping with 384 to 3,072 SNPs. SNPs in the three simulated panels were selected from the 50 k panel by compromising between uniform marker density and high MAF [23]. Number of SNPs on each chromosome is described in Table 1 for all panels.

**Table 1:** Number of SNPs on each chromosome for all SNP panels used in the study.

Chromosome	50 k	6 k	3 k	L1536	L768	L384
BTA1	2,291	369	160	97	49	24
BTA2	1,856	331	136	85	42	21
BTA3	1,792	284	120	77	38	19
BTA4	1,731	285	125	75	37	19
BTA5	1,504	283	117	76	38	19
BTA6	1,750	283	115	74	37	18
BTA7	1,510	260	108	68	34	17
BTA8	1,631	280	112	71	35	18
BTA9	1,427	255	108	65	33	16
BTA10	1,496	254	103	64	32	16
BTA11	1,590	268	104	67	33	17
BTA12	1,157	211	88	52	26	13
BTA13	1,236	206	86	51	25	13
BTA14	1,206	207	82	49	25	12
BTA15	1,200	202	83	51	25	13
BTA16	1,049	194	77	47	24	12
BTA17	1,137	185	72	46	23	12
BTA18	973	168	66	40	20	10
BTA19	977	163	57	39	20	10
BTA20	1,072	191	72	46	23	11
BTA21	947	174	73	42	21	10
BTA22	912	160	66	37	19	9
BTA23	800	146	52	32	16	8
BTA24	895	165	63	39	20	10
BTA25	746	136	46	26	13	7
BTA26	752	139	46	31	15	8
BTA27	714	131	47	30	15	7
BTA28	700	119	43	28	14	7
BTA29	739	128	53	31	16	8
Total	35,790	6,177	2,480	1,536	768	384

### Phenotypes

Milk yield, fat percentage, protein percentage, and somatic cell score (SCS) traits were considered for this study. Official bull proofs and reliabilities in April 2008 and December 2011 were obtained from Canadian Dairy Network (CDN). De-regressed proofs and corresponding reliabilities were derived by CDN from the 2008 bull proofs. Bulls born before 2004 and had reliabilities of de-regressed proofs greater than 0.80 were used as the training data set (n = 1,608). Bulls born in or after 2004 and had reliabilities of proofs greater than 0.80 in 2011 were used for validation (n = 3,232). Bulls born before 2004, but had less reliable de-regressed proofs in 2008 (n = 5,469) were used for genotype imputation only. Numbers of bulls in different groups are listed by birth year in Table 2.

**Table 2:** Number of bulls in different groups by birth year.

Year of birth	Reference^1^	Training	Validation
1950–1954	4	0	0
1955–1959	7	1	0
1960–1964	17	5	0
1965–1969	13	3	0
1970–1974	15	11	0
1975–1979	25	15	0
1980–1984	76	29	0
1985–1989	437	86	0
1990–1994	382	140	0
1995–1999	2,557	843	0
2000–2003	1,936	475	0
2004–2007	0	0	3,232
Total	5,469	1,608	3,232

1 Bulls in the reference group were used only for genotype imputation.

### Scenarios

Four scenarios were designed to mimic situations that different proportion of bulls in the training and validation sets were genotyped on low density panels. In scenario 0 (S0), all bulls (n = 10,309) had genotypes on the 50 k SNP panel. In scenario 1 (S1), all bulls in the training set had genotypes from the 50 k panel, and all bulls in the validation set (n = 3,232) had genotypes on low density panels. In scenario 2 (S2) and scenario 3 (S3), 33% and 66% of bulls were randomly selected from the training set, respectively, to have low density genotypes, and all bulls in the validation set had low density genotypes. Size of the reference population was also reduced correspondingly to have the same proportion of animals that had 50 k genotypes. Animals in the training set that had 50 k genotypes were also combined with the reference population for genotype imputation and thus the actual numbers of bulls in the reference population used for genotype imputation were 7,077 for S1, 4,741 for S2, and 2,406 for S3.

Genotype imputation was implemented using software FImpute (version 2) developed by Sargolzaei et al. [24]. FImpute uses family imputation algorithm followed by population imputation steps based on a sliding window technique [25].

### Genomic prediction

A GBLUP method and a Bayesian method with a spike and slab mixture prior distribution for marker effects were used to predict direct genomic values (DGV) for bulls in the validation set. The GBLUP method used a genomic relationship matrix proposed by VanRaden [5] and was implemented via the GEBV software [26]. For the Bayesian method, the statistical model can be written as:
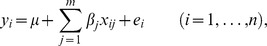
where *y_i_* is the phenotypic value (de-regressed EBVs) for animal *i*; *μ* is the population mean; *n* is the total number of animals, and *m* is the total number of SNPs; *x_ij_* is the genotype coded 0, 1 or 2 as number of copies from a randomly chosen allele; *β_j_* is the regression coefficient (allele substitution effect) for SNP *j*; and *e_i_* is the random residual effect.

The spike and slab mixture prior distribution for SNP effects is a mixture of two normal distributions with weights *π* and (1 - *π*), i.e., 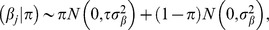 where *τ* is an arbitrarily small value. *π* was assigned a uniform prior distribution with a support on (0, 1). A scaled inverse Chi-square distribution with degree of freedom *v*_β_ and a scale 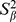 was assigned to 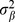. The strategy of using a group specific variance rather than a locus specific variance was to avoid the lack of Bayesian learning as in the BayesB method [1], [9], [27]. The arbitrarily small value *τ* shrinks the SNP effects towards zero so that it can effectively, but not completely remove an irrelevant SNP from the model. Advantages of using such a two-group mixture distribution have been discussed previously [28], [29]. *μ* was given a flat prior distribution, and the residual was assigned a normal distribution, i.e., 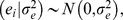 where 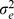 follows a scaled inverse Chi-square distribution with degree of freedom *v_e_* and a scale 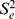.

A Gibbs sampling algorithm was developed to draw inferences from the joint posterior distribution. Hyper-parameters were determined before running Gibbs sampling. *v*_β_ and *v*_e_ were arbitrarily set to 4 and 10, respectively. *τ* was chosen to be 0.0001. The scale parameter 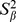 was derived from the expected value of a scaled inverse chi-square distributed random variable, i.e., 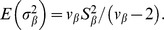 Assuming SNPs can capture all the additive genetic variance (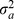), 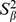 can be approximated by 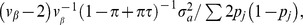 where *p_j_* is the allele frequency for SNP *j*. Similarly, 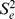 can be approximated by 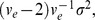 where 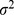 is the random environmental error variance. 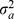 and 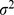 were estimated by ASReml 3.0 [30] from a preliminary analysis on de-regressed EBVs using an animal model. Residual effects were assumed to have a homogeneous variance due to a high cut-off value for the reliability of de-regressed EBVs in the training population.

A self-developed computer program was written with ANSI C language to implement the Gibbs sampler. The Gibbs sampling was run for 100,000 iterations with the first 20,000 cycles discarded as burn-in. Burn-in period were determined by visually inspecting the Gibbs sampling chain. All samples were kept after the burn-in and the sample means were used as estimates for SNP effects. Direct genomic breeding values for bulls in the validation set were estimated by summing the SNP effects over all loci.

### Evaluation

Imputed genotypes were compared to the actual genotypes from the 50 k panel and the percentage of genotypes imputed correctly out of the total imputed genotypes was calculated as a measure of imputation accuracy. For genomic prediction, Pearson's correlation coefficient between DGV and bull proofs from 2011 for bulls in the validation set was estimated as a measure of accuracy.

## Results and Discussion

### Accuracy of genotype imputation

Table 3 shows the accuracy of genotypes that were imputed from various low density panels to the 50 k SNP panel under different scenarios. The imputation accuracy was the highest (0.9841) when all bulls in the training set were genotyped with 50 k panel and bulls in the validation set were genotyped on the 6 k panel. Accuracy was dropped rapidly when the density of the SNP panels was decreased. Imputation accuracy from 6 k SNP panel were greater than that from the 3 k panel by about 2, 3 and 4 percentage points, when 0%, 33% and 66% of animals in the training set, respectively, were also genotyped on the low density panel. The accuracy decreased as more training bulls were genotyped on low density panels which, consequently, resulted in reduced reference size for imputation. The trends of imputation accuracy were consistent with reports from other studies [14], [22], [23].

**Table 3:** Accuracy of genotype imputation under different scenarios.

Scenario^1^	6 k	3 k	L1536	L768	L384
S1	0.9841	0.9604	0.9430	0.8787	0.7965
S2	0.9792	0.9507	0.9300	0.8573	0.7617
S3	0.9723	0.9367	0.9120	0.8285	0.7210

1 The reference population sizes used for imputation were n = 7,077, n = 4,741 and n = 2,406 for scenario S1, S2 and S3, respectively; 0%, 33%, and 66% of the training set in scenario S1, S2, and S3, respectively, and all bulls in the validation set were genotyped on the low density panel.

### Prediction accuracy using actual 50 k SNP genotypes

Table 4 shows the accuracy of DGV in the validation set predicted via the GBLUP and Bayesian methods using the observed 50 k SNP genotypes in the training and validation sets. The Bayesian method outperformed GBLUP by 3, 11, 5 and 0 percentage points, for milk yield, fat percentage, protein percentage and SCS, respectively, with the greatest difference for fat percentage and the least for SCS.

**Table 4:** Accuracy of genomic prediction and posterior estimates of π using observed 50 k SNP genotypes under scenario S0^1^.

Trait	Accuracy		Posterior π
	GBLUP	Bayesian	
Milk	0.61	0.64	0.96
Fat %	0.64	0.75	0.99
Protein %	0.71	0.76	0.99
SCS	0.62	0.62	0.93

1 S0: All animals in the training and validation sets were genotyped on the 50 k SNP panel.

Results also shown in the table were the posterior estimates of π, which were 0.96, 0.99, 0.99, and 0.93, for milk yield, fat percentage, protein percentage, and SCS, respectively. π is an indicator on the proportion of SNPs that were expected to have no effects on the trait. When π is small, the trait is likely affected by many QTL with small effects, and when π is large, a few large QTL are expected to influence the trait [9]. Daetwyler et al. [11] concluded that Bayesian methods would perform similarly or slightly worse than GBLUP when the trait was affected by many QTL each with a small effect, and better if the trait was influenced by a few large QTL. The largest π in our study was estimated for fat percentage, for which the Bayesian method showed the greatest advantage compared to GBLUP. A relatively smaller estimate of π for SCS corresponded to no difference of accuracy between the Bayesian and GBLUP methods. These results agreed with other studies comparing the performance between GBLUP and Bayesian methods for traits with different genetic architectures [12], [13].

**Figure 1:**
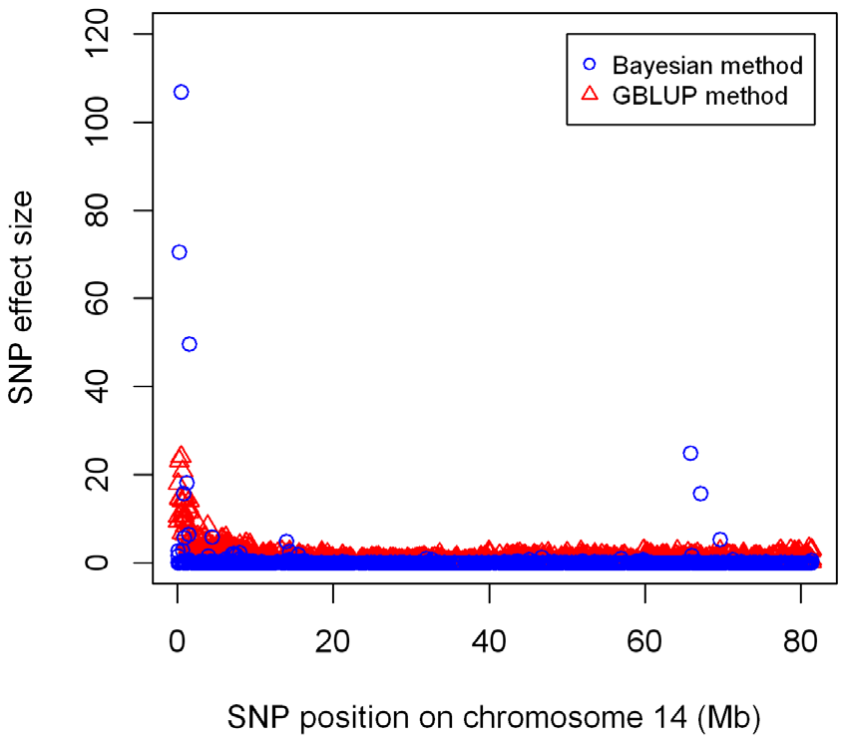
SNP effects for fat percentage estimated from GBLUP and the Bayesian methods.

To show the difference of estimated SNP effects between the GBLUP and Bayesian methods, Figure 1 plots estimated SNP effects on chromosome 14 for fat percentage. Both GBLUP and the Bayesian methods highlighted a QTL region harbouring the known DGAT1 gene which has been confirmed to have a large effect on milk fat percentage in cattle [31]. The Bayesian method tended to select fewer relevant SNPs, while the GBLUP method picked many more SNPs surrounding the QTL.

### Prediction accuracy using imputed 50 k SNP genotypes

Table 5 presents the accuracy of DGV for bulls in the validation set under different scenarios. Accuracies achieved from the Bayesian method were greater than or similar to those from GBLUP, for all low density panels, under all scenarios. Accuracy of genomic prediction decreased when the density in the SNP panel was reduced. For scenarios where genotype imputation subjected to more errors, accuracy of genomic prediction also declined more rapidly. The scenario S1used the same training population as scenario S0, and thus the estimated π values from the Bayesian method were the same as in S0. For scenario S2 and S3 in Table S1, the estimated π values were shown.

**Table 5:** Accuracy of genomic prediction using imputed 50: Accuracies from using Bayesian model are parenthesized and accuracies for GBLUP are presented outside the parenthesis.

Trait	Scenario^1^	Low density SNP panel				
		6 k	3 k	L1536	L768	L384
Milk	S1	0.61 (0.64)	0.60 (0.63)	0.59 (0.61)	0.55 (0.57)	0.49 (0.50)
	S2	0.61 (0.64)	0.59 (0.62)	0.58 (0.60)	0.52 (0.54)	0.44 (0.46)
	S3	0.61 (0.64)	0.59 (0.61)	0.57 (0.58)	0.50 (0.52)	0.39 (0.42)
Fat %	S1	0.64 (0.75)	0.63 (0.73)	0.59 (0.63)	0.54 (0.57)	0.42 (0.42)
	S2	0.64 (0.75)	0.63 (0.73)	0.56 (0.61)	0.50 (0.53)	0.37 (0.39)
	S3	0.64 (0.74)	0.62 (0.72)	0.53 (0.59)	0.45 (0.51)	0.32 (0.34)
Protein %	S1	0.71 (0.76)	0.70 (0.74)	0.69 (0.72)	0.65 (0.66)	0.57 (0.57)
	S2	0.71 (0.75)	0.69 (0.73)	0.68 (0.70)	0.62 (0.63)	0.53 (0.54)
	S3	0.71 (0.75)	0.68 (0.72)	0.67 (0.69)	0.58 (0.59)	0.47 (0.47)
SCS	S1	0.62 (0.62)	0.62 (0.62)	0.61 (0.61)	0.57 (0.57)	0.53 (0.53)
	S2	0.62 (0.62)	0.61 (0.61)	0.60 (0.60)	0.54 (0.55)	0.49 (0.50)
	S3	0.62 (0.61)	0.60 (0.60)	0.60 (0.60)	0.53 (0.54)	0.44 (0.46)

1 0%, 33%, and 66% of the training set in scenario S1, S2, and S3, respectively, and all bulls in the validation set were genotyped on the low density panel.

The trend that the accuracy changed with the density of SNP panel and with the proportion of training bulls being genotyped on the low density panels agreed with results of Weigel et al. [22]. However, in their study, only up to a 3 k low density panel was evaluated. Our study revealed that the 6 k SNP panel performed better than the 3 k panel and resulted in the least reduction of genomic prediction accuracy among all the low density panels. In fact, no reduction was observed for 6 k panel using GBLUP method under all scenarios, and only slight reductions (up to 1 percentage point) were observed for the Bayesian method under S2 and S3.

The influence of imputation errors on the genomic prediction accuracy also depends on the traits. For example, when bulls in the validation set were imputed from L384 SNP panel, accuracy of DGV predicted via GBLUP was reduced by a rate of 20, 34, 20 and 15%, for milk yield, fat percentage, protein percentage, and SCS, respectively; and for the Bayesian method, the accuracy was dropped by 22, 44, 25, and 15%, respectively. This was likely due to different genetic architectures underlying different traits. For traits affected by a few large QTL, such as fat percentage, accuracy of genomic prediction seemed much more sensitive to imputation errors than traits controlled by many small QTL, such as SCS. A similar trend was observed in a simulation study [32], where the accuracy of genomic prediction from low density panels declined much more rapidly for traits with a smaller number of QTL. Figure 1 showed that for chromosome regions with a large QTL, the Bayesian method tended to select fewer relevant SNPs, while GBLUP or the Ridge-regression picked many more SNPs surrounding the QTL. For other regions, the Bayesian method placed less weight on the SNPs than GBLUP. Therefore, the Bayesian method could suffer more if the few relevant SNPs were imputed with error, but the GBLUP method would suffer from imputation errors accumulated over many more SNPs. This could possibly explain why the Bayesian method had a greater reduction rate in accuracy for traits with large QTL and why the Bayesian method still resulted in higher or equal accuracies than GBLUP for all scenarios. Relative performance of the two methods might be more related to distributions of imputation errors. If more imputation errors were distributed around the QTL, one could speculate that the Bayesian method would suffer more from these errors than GBLUP and consequently resulted in more reductions in the accuracy of genomic prediction. Most of the economically important traits in dairy cattle may be controlled by large number of QTL with small effects, and the GBLUP and Bayesian method would perform similar for genomic prediction for these traits [11]–[13]. It is expected that for most of the complex traits the impact of imputation errors on the GBLUP and Bayesian method will be similar as observed for SCS in this study.

To examine the imputation errors on SNPs with large effects, two SNPs within the DGAT1 gene region were chosen. The two SNPs had the largest effects estimated from both the GBLUP and Bayesian methods. Imputation accuracies under scenario S1 are shown in Table 6. Both SNPs are present in the 6 k panel and thus the accuracies were 1. Imputation from the 3 k panel reached a higher accuracy than the average as shown in Table 3. But for L1536, L768, and the L384 panels, imputation accuracies for the two SNPs were below average. This could explain the rapid decline in accuracy of genomic prediction to 0.63 from 0.75 for the low density panel L1536 although the average imputation accuracy was not low (0.9430). To further investigate the potential of selecting SNPs with largest effects in the design of low density panels, actual genotypes from the two SNPs were included for genomic prediction under scenario S1 together with other imputed genotypes from the low density panels. Table 7 shows the results of genomic prediction using the GBLUP and Bayesian methods. Accuracy was increased by up to 2 percentage points for the GBLUP method. But for the Bayesian method, accuracies were increased by 1, 10, 13, and 22 percentage points, respectively, for the 3 k, L1536, L768, and L384 panels. These results suggest that a low density panel comprising SNPs with largest effects has the potential to preserve the accuracy of genomic prediction from higher density panels.

**Table 6:** Imputation accuracy under scenario S1 for two SNPs with largest effects on fat percentage.

SNP ID	Location^1^ (Bp)	Low density SNP panel				
		6 k	3 k	L1536	L768	L384
ARS-BFGL-NGS-4939	443,937	1	0.9830	0.8688	0.8181	0.7249
ARS-BFGL-NGS-57820	226,532	1	0.9802	0.8642	0.8165	0.7200

Bulls in the training were genotyped on the 50 k panel, and bulls in the validation set were genotyped on low density panels.

1 Locations of SNPs are shown as from the bovine genome assembly Btau4.2.

**Table 7:** Accuracy of genomic prediction under scenario S1 for fat percentage by including actual genotypes from the two SNPs with largest effects into the low density SNP panel.

Method	Low density SNP panel				
	6 k	3 k	L1536	L768	L384
GBLUP	0.64	0.63	0.60	0.56	0.44
Bayesian	0.75	0.74	0.73	0.70	0.64

Bulls in the training were genotyped on the 50 k panel, and bulls in the validation set were genotyped on low density panels.

Using either the GBLUP or Bayesian method, the 6 k SNP panel performed better than the 3 k panel. In this study, genotypes on both the 3 k and 6 k panel were simulated from the 50 k genotypes. In reality, there are more genotyping errors in 3 k genotypes than in 6 k or 50 k due to the Golden Gate genotyping technology used for the 3 k panel [14]. So, results could be worse for the 3 k panel in practice. The other lower density panels performed worse due to more inaccurate imputation of SNP genotypes. Accuracy for either genotype imputation or genomic prediction was evaluated as an average for the population. Examining imputation accuracies at an individual animal level showed that even for very low density SNP panels, there were still a substantial number of bulls that achieved high imputation accuracies. These animals should have close relationships to the reference set which were used by FImpute. In results not shown above, when both parents were genotyped on the 50 k SNP panel, accuracy of imputation from L384 SNP panel to 50 k was 0.9436. The imputation accuracy was 0.7738 when only sires were genotyped on the 50 k panel, and 0.6213 when no parents had 50 k genotypes. Zhang and Druet [23] also found that when the genomes of target animals were fully inherited from reference animals, imputation errors from a 384 SNP panel to 50 k SNP panel can be as low as 3.2%. In this study, imputed genotypes were included in the training set regardless of their imputation accuracies. In practice, only animals that have their genotypes imputed with high accuracies should be included in the training set. This scenario should be further studied to investigate the impact of using imputed genotypes on the accuracy of genomic prediction.

In the future, more and more animals might be genotyped on low density panels. One might have to decide whether to include these animals in the training population to derive genomic prediction equations. From Table 5, the accuracy of genomic prediction was consistently reduced when more animals in the training set were imputed when the density of the SNP panel was lower than 6 k. For the 6 k panel, accuracy of genomic prediction was almost not altered even 66% of the training set was imputed. This of course, was achieved by the high genotype imputation accuracy for the 6 k panel. Currently, nearly all males used for breeding are genotyped or re-genotyped on panels with a density of 6 k or higher and results from this study justified the application of the 6 k panel. In this study, the sample size of the training set was kept constant regardless the use of imputation. The use of imputation could, however, also aid to enlarge the sample size of the training set, which might in fact increase the accuracy of the DGV. This possible scenario warrants further investigation. This study only uses bull genotypes for genomic evaluations. Large numbers of females in dairy cattle have also been genotyped on the 50 k or low density panels. Although cautions have been taken to include cow genotypes in the training population currently due to biases in genomic evaluations caused by preferential treatment of elite cows [33], the problem can be alleviated by including genotypes of randomly selected cows [33] or by appropriately adjusting for cow evaluations [34]. A large percent of the cows may be genotyped cost-effectively on low density SNP panels followed by imputing to the 50 k genotypes. Including imputed genotypes from cows in the training population and its impact on the genomic predictions require further evaluation.

## Conclusions

Performance of both the Bayesian and GBLUP methods was influenced by imputation errors. For the traits considered in this study, the Bayesian method performed similar or better than the GBLUP method for genomic prediction under all scenarios and with different densities of the SNP panel or proportions of the training set being imputed. However, for traits affected by a few large QTL, the Bayesian method resulted in greater reductions of accuracy than GBLUP when a very low density of SNP panel was used. Including SNPs with largest effects in the low density panel substantially improved the accuracy of genomic prediction for the Bayesian method. When different low density panels were compared, with all animals in the training set genotyped on 50 k SNP panel, and animals in the validation set genotyped on the 6 k SNP panel, the accuracy of genomic prediction was the greatest for both GBLUP and the Bayesian methods. Imputation from SNP panels with a density lower than 6 k was more prone to errors and resulted in lower accuracy of genomic prediction. But for animals that have close relationship to the reference set, genotype imputation may still achieve a relatively high accuracy. Including genotypes imputed from the 6 k panel achieved almost the same accuracy of genomic prediction as that of the 50 k panel, even when 66% of the training animals were genotyped on the low density panels. These results justified the application of the 6 k panel for genomic prediction.

## Supporting Information

Table S1 **Estimated π values from the Bayesian model under scenario S2 and S3.**
(DOCX)
