# Supplementary material for: Impact of Genotype Imputation on the Performance of GBLUP and Bayesian Methods for Genomic Prediction
Source: PLoS One. 2014 Jul 15;9(7):e101544. doi: 10.1371/journal.pone.0101544 (PMC4099124; doi:10.1371/journal.pone.0101544)
Supplement: Table S1 — Estimated π values from the Bayesian model under scenario S2 and S3. (DOCX) [file pone.0101544.s001.docx]

### Table S1. Estimated π values from the Bayesian model under scenario S2 and S3.

| Trait | Low density SNP panel | | | | |
| --- | --- | --- | --- | --- | --- |
|  | 6k | 3k | L1536 | L768 | L384 |
| Scenario S2 | | | | | |
| Milk | 0.96 | 0.96 | 0.95 | 0.96 | 0.95 |
| Fat % | 0.99 | 0.99 | 0.99 | 0.99 | 0.99 |
| Protein % | 0.99 | 0.98 | 0.98 | 0.98 | 0.98 |
| SCS | 0.94 | 0.94 | 0.94 | 0.92 | 0.95 |
| Scenario S3 | | | | | |
| Milk | 0.95 | 0.94 | 0.94 | 0.94 | 0.95 |
| Fat % | 0.99 | 0.99 | 0.99 | 0.99 | 0.99 |
| Protein % | 0.99 | 0.98 | 0.98 | 0.98 | 0.98 |
| SCS | 0.94 | 0.92 | 0.92 | 0.89 | 0.93 |
